# Surgical Outcomes of Thyroid Nodules Positive for Gene Expression Alterations Using ThyroSeq V3 Genomic Classifier

**DOI:** 10.3390/cancers15010049

**Published:** 2022-12-22

**Authors:** Samer Salameh, Mohannad Rajab, Veronique-Isabelle Forest, Marc Pusztaszeri, Richard J. Payne

**Affiliations:** 1 Faculty of Medicine and Health Sciences, McGill University, Montreal, QC H3G 2M1, Canada; 2 Department of Otolaryngology Head & Neck Surgery, Jewish General Hospital, Montreal, QC H3T 1E2, Canada; 3 Department of Otolaryngology Head & Neck Surgery, McGill University Health Centre, Montreal, QC H4A 3J1, Canada; 4 Department of Pathology, Jewish General Hospital, Montreal, QC H3T 1E2, Canada

**Keywords:** thyroid nodule, thyroid neoplasm, molecular testing, ThyroSeq V3, gene expression alterations

## Abstract

**Simple Summary:**

This original research article aims to clarify the clinical and pathological features of thyroid nodules that express a specific category of genetic alterations found on molecular testing, known as gene expression alterations (GEAs). Using a sample of patients with thyroid nodules at two McGill University teaching hospitals in Montreal, Canada, this study shows that GEA is a potentially effective tool for diagnosing thyroid cancer and deciding between surgical versus non-surgical management of thyroid nodules.

**Abstract:**

ThyroSeq V3 (TsV3) tests for various genetic alterations, including gene expression alterations (GEAs), to improve diagnostic accuracy and clinical decision-making for indeterminate thyroid nodules. This study aimed to clarify the clinico-pathological features and outcomes of GEA-positive thyroid nodules, which have not yet been well-described in the literature. A retrospective chart review was performed whereby patients were included if they underwent thyroid surgery between January 2018 and May 2022 at two McGill University teaching hospitals and their surgery was preceded by pre-operative molecular TsV3 testing. In total, 75 of the 328 patients with thyroid nodules (22.9%) who underwent molecular testing and surgery were GEA-positive. On surgical pathology, GEA-positive nodules showed a significantly higher malignancy rate compared to their GEA-negative counterparts (90.7% vs. 77.7%, respectively, *p* = 0.011). Among those that were malignant, 48.5% had at least one aggressive pathological feature, including histological subtype, extra-thyroidal extension, or lymph node metastasis. *BRAF V600E* mutation had a significantly greater association with aggressive malignant GEA-positive nodules compared to non-aggressive ones (*p* < 0.001). This study demonstrates that GEA may be an effective diagnostic and prognostic tool for thyroid nodule management. However, further investigation is needed to characterize the clinico-pathological features of GEA in isolation and in association with other gene alterations.

## 1. Introduction

Thyroid cancer incidence rates have been increasing over the past few decades in many high-income countries, including Canada and the United States [1,2]. Differentiated thyroid cancers, such as papillary thyroid carcinoma (PTC) and follicular thyroid carcinoma (FTC), account for more than 90% of thyroid malignancies [3]. Thyroid ultrasound is a common initial diagnostic tool for thyroid nodules, which is followed by ultrasound-guided fine needle aspiration (USFNA) in select cases [4,5,6]. USFNA cytology provides a definitive diagnosis of benign or malignant nodules in 70–75% of cases, whereas the remainder of aspirates falls into one of three categories of indeterminate or suspicious cytology defined by the Bethesda system [7,8,9,10,11]. These include Bethesda categories III, IV, and V, with the expected risk of cancer at 5–15%, 20–30%, and 50–75%, respectively [8,9]. The uncertainty in cancer risk in these nodules precludes the optimal medical or surgical management of these patients and many of them undergo diagnostic surgery, which could be avoided in many patients with benign nodules [7,8,9,10,11]. 

Over the last decade, molecular testing has been increasingly used to improve the diagnosis and optimize the management of patients with thyroid nodules that carry an indeterminate cytological diagnosis, in addition to optimizing the surgical management of Bethesda category V and VI nodules [11,12]. Driver mutations such as *BRAF V600E* or *RAS* promote cancer development and are identified in more than 90% of thyroid cancers [3]. Other mutations such as *TP53* or *TERT* promoter drive the progression from differentiated to poorly differentiated or undifferentiated cancers, which are associated with aggressive behavior and short median time of survival [3,13,14]. One of the most commonly used molecular tests for indeterminate thyroid nodules is ThyroSeqV3 (TsV3) [15]. TsV3 is a 112-gene, DNA and RNA-based, targeted next-generation sequencing assay that tests for five classes of genetic alterations: (i) point mutations, (ii) indels, (iii) gene fusions (GF), (iv) copy number alterations (CNAs), and (v) gene expression alterations (GEAs) [15]. GEA studies are performed by comparing messenger RNA expression, via next-generation sequencing, detected in a thyroid USFNA against a panel of 167 genes [16]. Of these 167 genes, 142 are involved in an algorithm that identifies a benign gene expression pattern and the other 25 genes are involved in filtering out rare neoplasms and assessing for *BRAF V600E* mutations [16]. Investigations have demonstrated that GEAs of thyroid follicular cells potentially underlie the etiology of most well-differentiated thyroid cancers [13,14,15,16,17,18]. Therefore, identifying the gene expression patterns of transcripts could correlate the signaling pathways with the clinical presentation of disease and prognosis.

Accordingly, it is hypothesized that when GEA helps to rule out malignancy, it potentially obviates the need for diagnostic thyroidectomy in patients with indeterminate USFNA results [16,19]. Similarly, when GEA rules in a malignancy, it potentially assists in deciding the extent of surgery—total thyroidectomy versus hemithyroidectomy. However, the clinico-pathological features of GEA-positive thyroid nodules have not yet been well described in the literature. The objective of this study was to further clarify the clinico-pathological features and outcomes of GEA-positive thyroid nodules by examining a large cohort of patients that underwent molecular testing with TsV3 and subsequent surgical resection.

## 2. Materials and Methods

This study was approved by the McGill University Health Centre Research Ethics Board (ref # 2023-8845) in accordance with the Canadian Tri-Council Policy Statement of Ethical Conduct for Research Involving Humans.

A retrospective chart review was performed at the Jewish General Hospital (JGH) and McGill University Health Centre (MUHC) in Montreal, Quebec. Patients were included in the study if they had undergone thyroid surgery at the JGH or MUHC between January 2018 and May 2022 and had undergone pre-operative molecular testing with TsV3 [11,20]. Patients who were awaiting surgery or with no available surgical pathology at the time of data collection were excluded. The total number of patients included in this study was 328, including 75 patients who tested positive for GEAs and were categorized into a “GEA-positive” cohort and the remaining 253 patients who were categorized into a “GEA-negative” cohort. Patient characteristics, including sociodemographic information (age and sex) and oncologic characteristics (pre-operative ultrasound and cytology, molecular testing mutations/alterations, and post-operative histopathology) were recorded.

### 2.1. Tumor Analysis

Two USFNA nodule samples were collected for each patient using USFNA and placed into the same tube. Part of the total sample was transported to a commercial laboratory at the University of Pittsburgh Medical Center for TsV3 molecular testing. These samples were analyzed for molecular alterations, including genetic mutations and GEAs. The other part of the sample was sent to the pathology department at the affiliated hospitals for typical cytopathological analysis, and a Bethesda category was assigned according to the Bethesda system for reporting thyroid cytopathology [8,9,21].

### 2.2. Pathology

Final surgical specimens were reported by an experienced thyroid pathologist and classified according to the 2017 WHO classification of thyroid tumors [22]. Based on post-operative histopathology, nodules were placed under one of two categories: benign disease or malignant disease. Non-invasive follicular thyroid neoplasms with papillary-like nuclear features (NIFTP) were included in the malignant category since these low-risk neoplasms require conservative surgery for diagnostic and therapeutic purposes and share similar molecular alterations with their invasive counterpart [22]. Malignant tumors were classified as aggressive if they demonstrated at least one of the following pathological features: extra-thyroidal extension (ETE), lymph node metastasis (LN+), or aggressive histological subtype (columnar cell, tall cell, hobnail, solid/trabecular PTC, or poorly differentiated thyroid carcinoma [PDTC]).

### 2.3. Statistical Analyses

Two-way independent t-tests were used to compare continuous patient characteristics (i.e., age) between GEA-positive and -negative patient groups. Categorical patient and tumor characteristics (i.e., sex, nodule laterality, nodule size, Bethesda category, malignancy, malignancy type, aggressive features, and associated mutations/alterations) were analyzed using Pearson’s Chi-squared tests. Following univariate analyses, multivariate analysis was performed using a binomial logistic regression model to analyze the association between malignancy and the following covariates: GEA status, age, sex, and nodule size. The goodness-of-fit of the multivariate model was verified using the Hosmer–Lemeshow test. All statistical analyses were performed using commercially available software (SPSS Statistics version 27.0).

## 3. Results

A total of 328 patients (253 GEA-negative and 75 GEA-positive) were included in the study. On average, GEA-positive patients were diagnosed at a significantly younger age (46.8 vs. 53.7 years, respectively, *p* = 0.034). Both cohorts had similar proportions of female patients (80% positive vs. 79.8% negative, *p* = 0.964). GEA-positive and -negative thyroid nodules had similar proportions of right-sided laterality (56% vs. 56.3%, respectively, *p* = 0.957). The patient sociodemographic characteristics for both cohorts are presented in Table 1.

GEA-positive nodules had significantly larger proportions of higher-risk Bethesda categories (21.3% category III, 34.7% category IV, 16% category V and 28% category VI) compared to GEA-negative nodules (48.8% category III, 30.4% category IV, 14.4% category V and 4.4% category VI; *p* < 0.001). Moreover, GEA-positive nodules comprised 11.6% of all Bethesda III nodules, 25.5% of all Bethesda IV nodules, 25% of all Bethesda V nodules, and 65.6% of all Bethesda VI nodules. GEA-negative nodules showed a slightly higher mean nodule size in cm compared to GEA-positive nodules (2.6 vs. 2.1, respectively, *p* = 0.06). GEA-positive nodule size ranged from 0.6 cm to 6.2 cm. On surgical pathology, 90.7% of GEA-positive nodules were malignant, showing a significantly higher malignancy rate compared to their GEA-negative counterparts (90.7% vs. 77.9%, respectively, *p* = 0.011). Indeterminate GEA-positive nodules (Bethesda III and IV) showed a higher malignancy than their GEA-negative counterparts but this was non-significant (83.3% vs. 74.2%, respectively, *p* = 0.211). Malignant GEA-positive nodules showed a significantly higher aggressivity rate compared to their GEA-negative counterparts (48.5% vs. 23.4%, respectively, *p* < 0.001) The proportion of malignancy type did not differ significantly between GEA-negative and -positive nodules (*p* = 0.646). In the GEA-negative cohort, 80.2% of malignant cases were PTCs (158/197), 6.6% were FTCs (13/197), 3.6% were Hürthle cell (oncocytic) carcinoma (HCCs) (7/197), 7.6% were NIFTP (15/197), and 2% were PDTC (4/197). In the GEA-positive cohort, 88.2% of malignant cases were PTCs (60/68), 4.4% were FTCs (3/68), 2.9% HCCs (2/68), 2.9% were NIFTP (2/68), and 1.5% were PDTC (1/68). Patient primary oncologic characteristics for both cohorts are summarized in Table 1.

On multivariate analysis, GEA positivity was found to be significantly associated with malignancy (OR 2.84, 95% CI 1.21–6.68) when adjusting for age, sex, and nodule size. On the other hand, age (OR 1.00, 95% CI 0.98–1.02), sex (OR 0.70, 95% CI 0.36–1.35), and nodule size (OR 1.00, 95% CI 0.83–1.20) were not significantly associated with malignancy. The results of our multivariate model are summarized in Table 2.

In malignant GEA-positive nodules, there was an equal amount of multifocal (*n* = 34) and unifocal (*n* = 34) tumors. Furthermore, 48.5% of malignant GEA-positive nodules had at least one aggressive feature, as described previously in the final pathology report. In total, 23.5% (16/68) of malignant GEA-positive nodules had lymph node metastasis, 10.3% (7/68) had an extra-thyroidal extension, and 1.5% (1/68) were PDTC. With regards to PTC variants, 13.3% (8/60) were classical variants, 48.3% (29/60) were follicular variants, 3.3% (2/60) were oncocytic variants, and 35% (21/60) were aggressive subtypes. Among the aggressive subtypes in PTC nodules, we observed tall cell (21.7%, 13/60), hobnail (3.3%, 2/60), and solid/trabecular (10%, 6/60) histology. Columnar histology was not observed. Malignant GEA-positive nodule features are summarized in Table 3.

Eighty-four percent (84%) of thyroid nodules with GEAs were associated with another molecular mutation or alteration. In thyroid nodules with aggressive features, GEA was most commonly associated with *BRAF V600E* mutation (*n* = 18, 54.5%) and CNAs (*n* = 5, 15.2%). Furthermore, *BRAF V600E* mutation had a significantly greater association with aggressive GEA-positive nodules compared to non-aggressive ones (54.5 % vs. 8.6%, respectively, *p* < 0.001). In thyroid nodules with no aggressive features, GEA was most commonly associated with the *HRAS* mutation (*n* = 12, 34.3%), *NRAS* mutation (*n* = 10, 28.6%), and CNA (*n* = 8, 22.8%). Furthermore, the *HRAS* mutation had a significantly greater association with non-aggressive GEA-positive nodules compared to aggressive (34.3% vs. 12.1%, respectively, *p* = 0.031), and similarly for *NRAS* mutation (28.6% vs. 6.1%, respectively, *p* = 0.015). There was no significant difference in CNA association between aggressive and non-aggressive GEA-positive nodules (15.1% vs. 22.8%, respectively, *p* = 0.419). GEA-associated mutations and alterations for aggressive and non-aggressive malignant thyroid nodules are summarized in Table 4.

GEA-positive nodules associated with *BRAF V600E* mutation were predominantly Bethesda category VI (76.2%) with an overall malignancy rate of 100%. GEA-positive nodules associated with the *HRAS* mutation were predominantly Bethesda category IV (70.6%) with an overall malignancy rate of 94.1%. GEA-positive nodules associated with the *NRAS* mutation were predominantly Bethesda categories III (53.8%) and IV (38.5%) and had an overall malignancy rate of 92.3%. GEA-positive nodules associated with CNA were most frequently in Bethesda category IV (57.1%) with an overall malignancy rate of 92.9%. Bethesda category proportions and malignancy rates of GEA-associated mutations/alterations are summarized in Table 5.

## 4. Discussion

Scant data are available on the clinico-pathological features of thyroid nodules positive for GEA alterations using the TsV3 genomic classifier. In the original validation study for indeterminate thyroid nodules by Steward et al, the prevalence of GEAs in test-positive samples was 8% (*N* = 8) with a malignancy/NIFTP rate of 75%, including classic PTC (37%), NIFTP (13%), and other cancers including medullary thyroid carcinoma and metastatic renal cell carcinoma (25%) [21]. A recent study by our group demonstrated a GEA nodule malignancy rate of 100% and an aggressivity rate of 36.8% from a total sample size of 19 nodules [20]. To the best of our knowledge, the current study represents the largest number of GEA-positive thyroid nodules with surgical follow-up. The results demonstrate that TsV3 GEA-positive thyroid nodules that underwent surgical excision are significantly more likely to be malignant in comparison to nodules without GEA expression, with an overall malignancy rate of 90.7% and aggressivity rate of 48.5%. The association between GEAs and malignancy remains significant when controlling for age, sex, and nodule size on multivariate analysis. One key difference between the two other studies, however, was that 100% of GEA nodules were designated as Bethesda III or IV, while our study included a considerable proportion of Bethesda V and VI nodules (Table 1) [20]. When we compare the malignancy rates of indeterminate (Bethesda III and IV) nodules in both cohorts, GEA-positive nodules still have a higher malignancy rate (83.3% vs. 74.2%) but the difference is non-significant. It is also worth emphasizing that 84% of GEA-positive nodules in this study were associated with another mutation or alteration. Among the 12 thyroid nodules in our study that tested positive for GEA without any other mutation or alteration, 10 nodules (83.3%) were malignant, reflecting a similar malignancy rate to GEA-negative nodules in our study (77.5%); however, the sample size is too small to draw conclusions about the clinico-pathological characteristics of GEA alone. Expanding the patient population to a nationwide or multinational database may provide further insights into the aforementioned characteristics. 

A notable finding in our study was the association between GEA and three mutations/alterations: *BRAF V600E*, *HRAS*, and CNA. Unsurprisingly, the strongest association in GEA-positive nodules with aggressive malignant features was with *BRAF V600E*. *BRAF V600E* is the most frequent genetic mutation in PTC and was reported as a predictor of poor prognosis [6,23]. It is emphasized that the detection of *BRAF V600E* may drive toward total thyroidectomy if the clinic-pathological setting is appropriate [23,24,25,26,27]. As expected, the strongest association in GEA-positive nodules with no aggressive features was with *HRAS* and *NRAS* mutations. The low-risk nature of thyroid nodules with *RAS* and *RAS*-like mutations is well-established, as they are typically associated with Bethesda categories III and IV and a variety of follicular-pattern thyroid tumors, with most being minimally invasive and low risk [20,23,24,25,26,27,28]. *RAS* nodules often tend to be managed more conservatively with a lobectomy [28]. Nevertheless, the aforementioned associations with *BRAF V600E* and *HRAS* may be suggestive of an interactive role for GEA in the context of thyroid tumor microenvironments.

Previous studies have shown that thyroid tumor cells express a genomic profile different from that of normal thyroid cells [17,19,21]. At the molecular level, the miRNA–mRNA regulatory network was shown to have a functional relationship with tumor initiation and progression in several cancers, including PTC [13,14,15,16,17,18]. In particular, GEAs in adhesion molecules are linked to all stages of tumor progression, including tumor cell detachment, intravasation, and extravasation [13,14,15,16,17,18]. Studies have also demonstrated that signals from the thyroid tumor surrounding the microenvironment are integral to tumor initiation and growth [17,19,21,29]. The interplay between GEA gene dysregulation and pathways pertaining to cell metabolism, apoptosis, response to hypoxia, migration, and proliferation was demonstrated [17,19,21,29]. This is suggestive of a facilitating role for GEA in the context of thyroid malignancy, necessitating association with other mutations or alterations to dictate the pathway of tumor development. We find in our study that the proportions of thyroid cancer type (PTC, FTC, HCC) do not significantly differ between GEA-positive and -negative nodules. Although it was initially suggested that HCC demonstrated the most distinct GEA changes, more recent studies have shown that Hürthle cell and non-Hürthle cell cancer GEAs are largely similar [21,30]. The most notable differences in GEA are indeed found between *BRAF V600E* positive and negative PTCs. Consequently, characterizing the association between GEA and *BRAF V600E* mutations may be integral to refining the diagnosis, prognosis, and management of indeterminate thyroid nodules.

The guidelines delineated by the American Association of Endocrine Surgeons have deemed molecular testing a highly recommended diagnostic aid for cytologically indeterminate thyroid nodules [24,31,32,33]. Molecular techniques such as TsV3 enhance the management of thyroid nodules by identifying mutations highly specific for malignancy such as *BRAF V600E*, *RET* or *TERT*, ultimately reducing the number of diagnostic thyroidectomies required [24,31,32,33]. In particular, identifying a *BRAF V600E* mutation pre-operatively can help clinicians recognize potentially high-risk thyroid nodules and offer appropriate patient counseling and management. Without molecular testing, the management of indeterminate thyroid nodules often depends on choosing between a diagnostic lobectomy or active surveillance based on factors such as the nodule size, nodule growth, suspicious ultrasound features, family history, previous radiation exposure, and patient preferences [20,24,31,32,33]. The primary aim of our study was to determine the key clinicopathological features of thyroid nodules expressing GEAs. It was found that GEA-positive nodules that underwent surgical excision were associated with a significant increase in malignancy rate; however, this was predominantly in the context of associations with other mutations and alterations, including a strong association with *BRAF V600E* in aggressive malignancies. Consequently, subsequent investigations are warranted to further delineate the clinicopathological characteristics of GEAs in the absence of any other mutations and alterations. Furthermore, investigating the molecular and empirical relationship between GEAs and *BRAF V600E* may prove to be a vital piece in the coordinated effort to pre-operatively risk-stratify patients using molecular testing. This study provides an important first step toward utilizing GEAs to refine the ability of clinicians to predict malignancy and tumor aggressivity in thyroid nodules.

There are several limitations to this study that limit the interpretation of the results. A major limitation pertains to conclusions regarding the diagnostic accuracy of pre-operative GEA testing for indeterminate thyroid nodules. The inclusion of Bethesda VI nodules, which constitute 28% of GEA-positive nodules, serves the purpose of helping to characterize the clinicopathological features of all GEA-positive nodules. However, this has led to an increased observed malignancy rate of GEA-positive nodules; this is evident as the malignancy rate decreases when our analysis is restricted to indeterminate nodules. The malignancy rate also decreases when GEA-positive nodules have no other associated mutations/alterations; of note, the sample size of nodules with only GEAs is too small to draw conclusions. Moreover, GEA testing was also unable to detect the 77.9% of GEA-negative nodules which ended up becoming malignant. The aforementioned points highlight important avenues for future investigation in order to properly establish the utility of pre-operative GEA testing. This study provides an important first step by characterizing the clinico-pathological features of GEA-positive nodules. Other limitations include the retrospective design of the study, which increases the likelihood of selection bias. The strict inclusion of patients who underwent both pre-operative TsV3 molecular testing as well as thyroid surgery is more likely to increase malignancy rates since it likely includes patients with more clinically suspicious thyroid tumors. The process of thyroid malignancy workup and treatment may have been affected by the variability of primary care center or physician on presentation, comorbidities, and tertiary care center referrals. The limited financial access to molecular testing must also be considered, as it is not always covered by public healthcare insurance plans in Quebec.

## 5. Conclusions

This study demonstrates that 90.7% of thyroid nodules that underwent surgery with GEAs are malignant and 48.5% of those that are malignant have aggressive features. GEA-positive nodules have a malignancy rate of 92.1% when associated with another mutations/alterations, and a malignancy rate of 83.3% when not associated with another mutations/alterations. Thyroid nodules with GEAs are significantly more likely to have aggressive features if they are associated with the *BRAF V600E* mutation. Thyroid nodules with GEA and low-risk mutations such as *HRAS* or *NRAS* are significantly more likely to be low-grade malignancies such as a follicular variant of papillary thyroid carcinoma. Although our study highlights the major clinico-pathological features of thyroid nodules with GEAs, further investigation is needed to characterize GEAs in isolation and in association with *BRAF V600E* in order to properly assess their diagnostic and prognostic utility.

## Figures and Tables

**Table 1:** Sociodemographic and Oncologic Characteristics of Thyroid Nodule Patients with and without Gene Expression Alterations.

Patient & Nodule Characteristics	GEA Negative (*n* = 253)	GEA Positive (*n* = 75)	*p*-Value
Mean Age at Surgery (range)	53.7 (25–88)	46.8 (16–78)	0.034 *
Female, *N* (%)	201 (79.8)	60 (80.0)	0.964
Nodule Laterality, Right, *N* (%)	142 (56.3)	42 (56)	0.957
Bethesda Score Distribution, *N* (%)			<0.001 *
*III*	122 (48.8)	16 (21.3, 11.6) ^#^	
*IV*	76 (30.4)	26 (34.7, 25.5) ^#^	
*V*	36 (14.4)	12 (16, 25) ^#^	
*VI*	11 (4.4)	21 (28, 65.6) ^#^	
Mean Nodule Size in cm (range)	2.6 (0.7–11.7)	2.1 (0.6–6.2)	0.06
Malignant Nodules, *N* (%)	197 (77.9)	68 (90.7)	0.011 *
Malignant Indeterminate Nodules, *N* (%) ^A^	147 (74.2)	35 (83.3)	0.211
Any Aggressive Features, *N* (%)	46 (23.4)	33 (48.5)	<0.001 *
Malignancy Type, *N* (%)			0.646
Papillary Thyroid Carcinoma	158 (80.2)	60 (88.2)	
Follicular Thyroid Carcinoma	13 (6.6)	3 (4.4)	
Hürthle Cell Carcinoma	7 (3.6)	2 (2.9)	
NIFTP	15 (7.6)	2 (2.9)	
PDTC	4 (2.0)	1 (1.5)	

* Denotes statistical significance (*p* < 0.05); ^#^ (Percentage of GEA-positive nodules, Percentage of all nodules in Bethesda Category); ^A^ Percentage calculated out of number of indeterminate (Bethesda III and IV) nodules in each cohort; Abbreviations: Gene Expression Alterations (GEA), Non-invasive follicular thyroid neoplasm with papillary-like nuclear features (NIFTP), Poorly Differentiated Thyroid Carcinoma (PDTC).

**Table 2:** Binomial Logistic Regression of Association between Covariates and Malignancy.

Covariates	OR (95% CI)	*p*-Value	Hosmer–Lemeshow Goodness-of-Fit *p*-Value
**Gene Expression** **Alterations**	2.84 (1.21–6.68)	0.017 *	0.664
**Age**	1.00 (0.98–1.02)	0.926	
**Sex (Female)**	0.70 (0.36–1.35)	0.281	
**Nodule Size**	1.00 (0.83–1.20)	0.970	

* Denotes statistical significance (*p* < 0.05).

**Table 3:** Oncologic Features of Malignant Gene Expression Profile Positive Thyroid Nodules.

	Malignant GEA Nodules (*n* = 68)
Tumor Focality, *N* (%)	
Multifocal	34 (50)
Unifocal	34 (50)
Any aggressive features, *N* (%)	33 (48.5)
Aggressive features, *N* (%)	
ETE	7 (10.3)
LN+	16 (23.5)
PDTC	1 (1.5)
PTC Variant, *N* (%)	**PTC Nodules (*n* = 60)**
Classical	8 (13.3)
Follicular	29 (48.3)
Oncocytic	2 (3.3)
Other (Aggressive)	21 (35)
Aggressive PTC Histology, *N* (%)	
Tall cell	13 (21.7)
Columnar	0
Solid/Trabecular	6 (10)
Hobnail	2 (3.3)

Abbreviations: Gene Expression Alterations (GEA), Extra-Thyroidal Extension (ETE), Lymph Node Metastasis (LN+), Poorly Differentiated Thyroid Carcinoma (PDTC), Papillary Thyroid Cancer (PTC).

**Table 4:** Associated Mutations and/or Alterations within Gene Expression Alteration-Positive Thyroid Nodules.

Associated Mutations/Alterations, *N* (%)	Aggressive Nodules (*n* = 33)	Non-Aggressive Nodules (*n* = 35)	*p*-Value
***BRAF V600E***	18 (54.5)	3 (8.6)	<0.001 *
***HRAS***	4 (12.1)	12 (34.3)	0.031 *
***NRAS***	2 (6.1)	10 (28.6)	0.015 *
***CNA***	5 (15.2)	8 (22.8)	0.419
***THADA/IGF2BP3***	0 (0)	2 (5.7)	-
***EIF1AX***	2 (6.1)	0 (0)	-
***TERT***	2 (6.1)	0 (0)	-
***Other***	4 (12.1)	2 (5.7)	-

* Denotes statistical significance (*p* < 0.05).

**Table 5:** Bethesda Score Proportions and Malignancy Rates of Gene Expression Alteration-Associated Mutations and Alterations.

Mutation/Alteration, *N* (%)	B–III	B–IV	B–V	B–VI	Malignant
*BRAF V600E* (*n* = 21)	2 (9.5)	0 (0)	3 (14.3)	16 (76.2)	21 (100)
*HRAS* (*n* = 17)	2 (11.8)	12 (70.6)	3 (17.6)	0 (0)	16 (94.1)
*NRAS* (*n* = 13)	7 (53.8)	5 (38.5)	1 (7.7)	0 (0)	12 (92.3)
CNA (*n* = 14)	2 (14.3)	8 (57.1)	2 (14.3)	2 (14.3)	13 (92.9)

Abbreviations: Bethesda III (B–III), Bethesda IV (B–IV), Bethesda V (B–V), Bethesda VI (B–VI).

## Data Availability

The datasets used and/or analyzed during the current study are available from the corresponding author upon reasonable request. The datasets are not publicly available due to confidentiality and data security guidelines at our tertiary care centers.
